# Phytotoxic effects of invasive *Ageratina adenophora* on two native subtropical shrubs in Nepal

**DOI:** 10.1038/s41598-021-92791-y

**Published:** 2021-07-01

**Authors:** Tej Bahadur Darji, Barsha Adhikari, Seeta Pathak, Shristi Neupane, Lal B. Thapa, Tara Datt Bhatt, Ramesh Raj Pant, Gunanand Pant, Khadka Bahadur Pal, Kiran Bishwakarma

**Affiliations:** 1 Central Department of Botany, Institute of Science and Technology, Tribhuvan University, Kathmandu, Nepal; 2 Central Department of Environmental Science, Institute of Science and Technology, Tribhuvan University, Kathmandu, Nepal; 3 Department of Plant Resources, Ministry of Forests and Environment, Government of Nepal, Thapathali, Kathmandu, Nepal; 4 Department of Biology, Kailali Multiple Campus, Tribhuvan University, Dhangadhi, Nepal; 5 Department of Chemistry, Tri-Chandra Multiple Campus, Tribhuvan University, Kathmandu, Nepal; 6 Institute of Tibetan Plateau Research, Chinese Academy of Sciences, Beijing, China

**Keywords:** Ecophysiology, Invasive species, Ecology, Plant sciences

## Abstract

The response of native plants to allelopathic interference of invasive species may differ from species to species. In this study, the phytotoxic effects of *Ageratina adenophora* were tested on two native shrubs (*Osbeckia stellata* and *Elsholtzia blanda*) of Nepal. Both the shrubs were grown in pots under treatments of *A. adenophora* fresh leaves and root leachates, and litter. Then, the seedling length and biomass were compared among the treatments. The results show that *A. adenophora* litter has stimulatory effects but the leachates from fresh leaves and root are phytotoxic to the growth and development of native shrubs. Infrared Spectroscopy (IR) analysis confirmed the presence of O–H (Hydroxyl), N–H (Amines), C≡C (Alkynes), and C–H stretching (Aromatic) or C–O–C stretching (Ethers) in the leachates representing harmful allelochemicals. The invaded soil by *A. adenophora* had low pH and a high amount of organic matter, total nitrogen, phosphorus, and potassium than the uninvaded soil. The results indicate that the native *O. stellata* and *E. blanda* are harmed by *A. adenophora* in nature by leaching of allelochemicals and probably by reducing the soil pH. Overall, this study has provided valuable insights regarding the effects of *A. adenophora* invasion on native shrubs and revealing the potential mechanism of its invasiveness.

## Introduction

*Ageratina adenophora* (Spreng.) R.M. King & H. Rob., a perennial shrub of family Asteraceae, is commonly called the Crofton weed. It is the native weed in Mexico^1^. It has become highly invasive and rapidly spread worldwide including Asia, Australia, and Africa^1–3^. In the invaded regions, it has threatened the biodiversity of native forests, rangelands, and farmlands^4^. This weed has the capability to regenerate by vegetative methods and also reproduce from its minute seeds which are produced in huge numbers. It proliferates rapidly in the invaded sites and forms its monoculture^5,6^.

*Ageratina adenophora* has been naturalized in Nepal where it was first reported in 1952^7^. In Nepal, it is locally called ‘*Kalo Banmara*’ meaning the 'Forest Killer Plant' having dark green leaves. It has been spreading throughout the country from tropical regions to northern border crossing through the subtropical mountain region^7^. It has been spread along the trails, roads, disturbed sites, and margins or open canopy areas of the forests of Nepal^8–14^.

This plant is known to have negative impacts on native vegetation^15–17^. It affects plant community composition, species diversity, and abundance^15^. Thapa et al. (2020) reported that the plant is responsible to reduce the native species richness in the invaded sites in Nepal ^17^. Allelopathy has been one of the mechanisms affecting other plants by *A. adenophora*^18^. Negative impacts on physiology and morphology of some crops (e.g., rice), weeds (e.g., *Lolium perenne*, *Trifolium repens*, *Galinsoga parviflora,* and *Medicago sativa*) and native trees (e.g., *Schima wallichii* and *Alnus nepalensis*) by aerial parts (leaves and litter) and roots of this species have been reported previously^16,19–22^. Negative effects on native seed germination and growth by volatile compounds from the litter of *A. adenophora* have also been reported ^17,23^. In the inhibition, there is role of several allelochemicals present in the aerial and underground parts of this plant ^23–25^. The allelochemicals are the secondary metabolites that can be classified according to their carbon skeletal structure and type of functional groups such as alcohols, amines, carboxylic acids^26^. Thus, the functional group analysis is one of the most satisfactory and applicable methods of determining organic compounds which also lends towards the identification of organic compounds^27^. Such analyses have significance in understanding the chemical nature of invasive weed’s allelochemicals.

Although the previous studies have documented the negative impacts of *A. adenophora* on the native species, the insights regarding whether *A. adenophora* affects all the native plants always negatively or it may also have positive impacts on some native species are very scarce. For this, several experiments can be performed by testing the effect of *A. adenophora* on the native plants from invaded sites. We hypothesized that the native plants’ response to allelopathic interference of *A. adenophora* differs from species to species. Some native species are found frequently associated with *A. adenophora* in the invaded sites*.* There might be two reasons for such an association: one, the invasive species might have started colonization in the native region and the native species might be at the stage of replacement/inhibition by the invasive species. Another, the native species might have resisted the negative effects of invasion and therefore, the association has no significant negative relationship or sometimes there might be a positive interaction between them. Such phenomena cannot be predicted easily by simple field observations.

Two of the native species (*Osbeckia stellata* and *Elsholtzia blanda*) in the mid-hill region of central Nepal are frequently associated with *A. adenophora* invaded forests. Both of these species are the most common subtropical native shrubs in Nepal. As discussed above, it cannot be expected that these two native species are either tolerating the negative effects of *A. adenphora* or they are harming by the invasion. In such a condition, proper experiments should be designed to know the actual interactions among them. In this study, growth response of above mentioned native shrubs were tested against *A. adenphora* fresh leaves and roots leachates, and the litter. In addition, the chemical groups present in the leachates and the potential impact of *A. adenophora* invasion in soil was also explored. Testing phytotoxicity of *A. adenophora* on native shrubs of Nepal is a novel work. It would have significance to know the parts and chemicals found in *A. adenphora* contributing positively or negatively to the growth and development of native shrub species and soil properties.

## Results

### Shoot and root length

**Figure 1:**
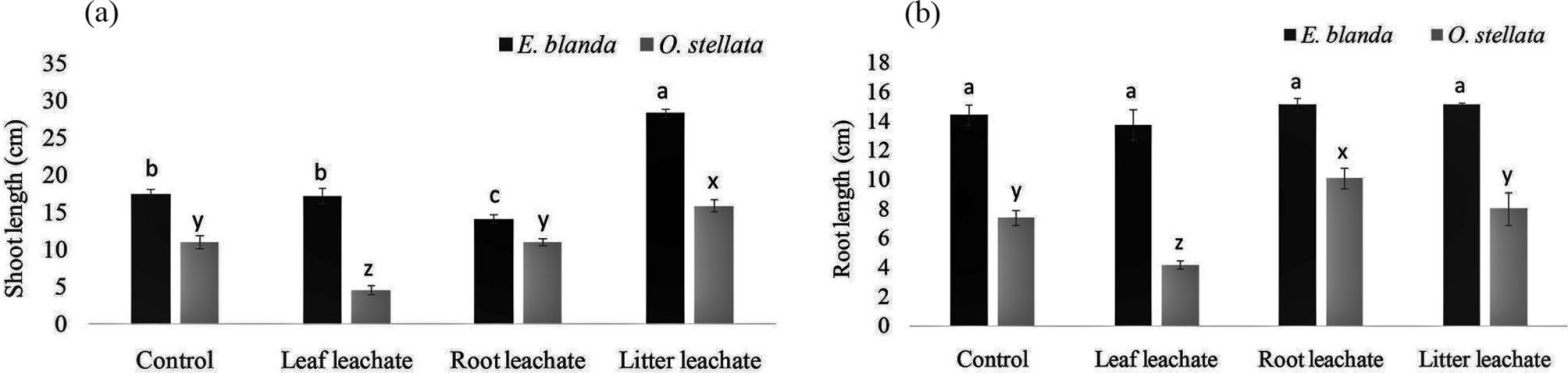
Effect of *A. adenophora* leachates on (**a**) shoot length and, (**b**) root length of native *E. blanda* and *O. stellata.* Different letters above the error bar indicate significant differences among the treatments (‘a’, ‘b’, and ‘c’ for *E. blanda;* ‘x’, ‘y’, and ‘z’ for *O. stellata; p* < 0.05).

Shoots of both the native species *E. blanda* and *O. stellata* were longer in *A. adenophora* litter treatment compared to the control plants. *A. adenophora* root leachate reduced the shoot length of *E. blanda* while the leaf leachate did not show inhibitory or stimulatory effects. Interestingly, the shoot length of *O. stellata* was inhibited by the fresh leaf leachate, not by the root leachate of *A. adenophora* (Fig. 1a, df = 3, *p* < 0.001).

In the case of root length, there was no stimulatory or inhibitory effects on *E. blanda* by all the treatments (Fig. 1b, df = 3, *p* = 0.44)*.* The roots of *O. stellata* were longer in the treatment of *A. adenophora* root leachate comparing to the control plants while the leaf leachate showed inhibition to the root length (Fig. 1b, df = 3, *p* < 0.001).

### Shoot and root dry weight (biomass)

**Figure 2:**
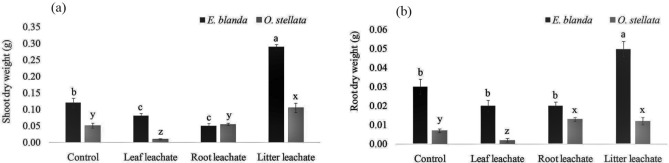
Effect of *A. adenophora* leachates on (**a**) shoot dry weight and (**b**) root dry weight of native *E. blanda* and *O. stellata.* Different letters above the error bar indicate significant differences among the treatments (‘a’, ‘b’, and ‘c’ for *E. blanda*; ‘x’, ‘y’, and ‘z’ for *O. stellata; p* < 0.05).

Similar to the shoot length, *A. adenophora* litter increased dry weight of shoots in both native species. Comparing to the control plants shoots were lighter in the treatments of *A. adenophora* leaf and root leachates. The root leachate did not inhibit the dry weight of *O. stellata* but the leaf leachate reduced the weight significantly (Fig. 2a, df = 3, *p* < 0.001).

The dry weight of roots in both the native species was increased by *A. adenophora* litter. There was no significant effects of both the leaf and root leachate on the root dry weight of *E. blanda.* The root leachate also increased dry weight of roots in *O. stellata* while the leaf leachate reduced the dry weight (Fig. 2b, df = 3, *p* < 0.001).

### Chlorophyll a and b

**Figure 3:**
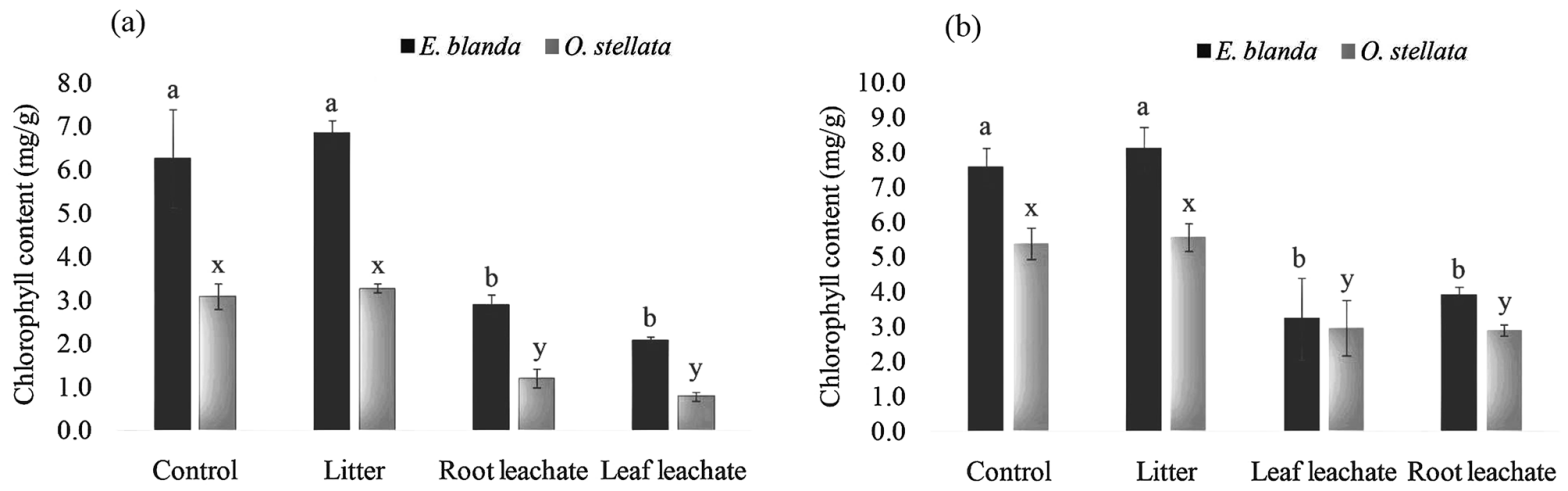
Effect of *A. adenophora* leachates on (**a**) chlorophyll - a and, (**b**) chlorophyll - b of native *E. blanda* and *O. stellata.* Different letters above the error bar indicate significant differences among the treatments (‘a’, ‘b’, and ‘c’ for *E. blanda*; and ‘x’, ‘y’, and ‘z’ for *O. stellata; p* < 0.05).

*Ageratina adenophora* litter did not increase or decrease the content of chlorophyll - a and b in both *E. blanda* and *O. stellata* but the leaf and root leachates inhibited chlorophyll content significantly [Fig. 3, df = 3, *p* < 0.01 (*E. blanda*) and *p* < 0.001 (*O. stellata*)].

### Infrared spectroscopy (IR) analysis

**Figure 4:**
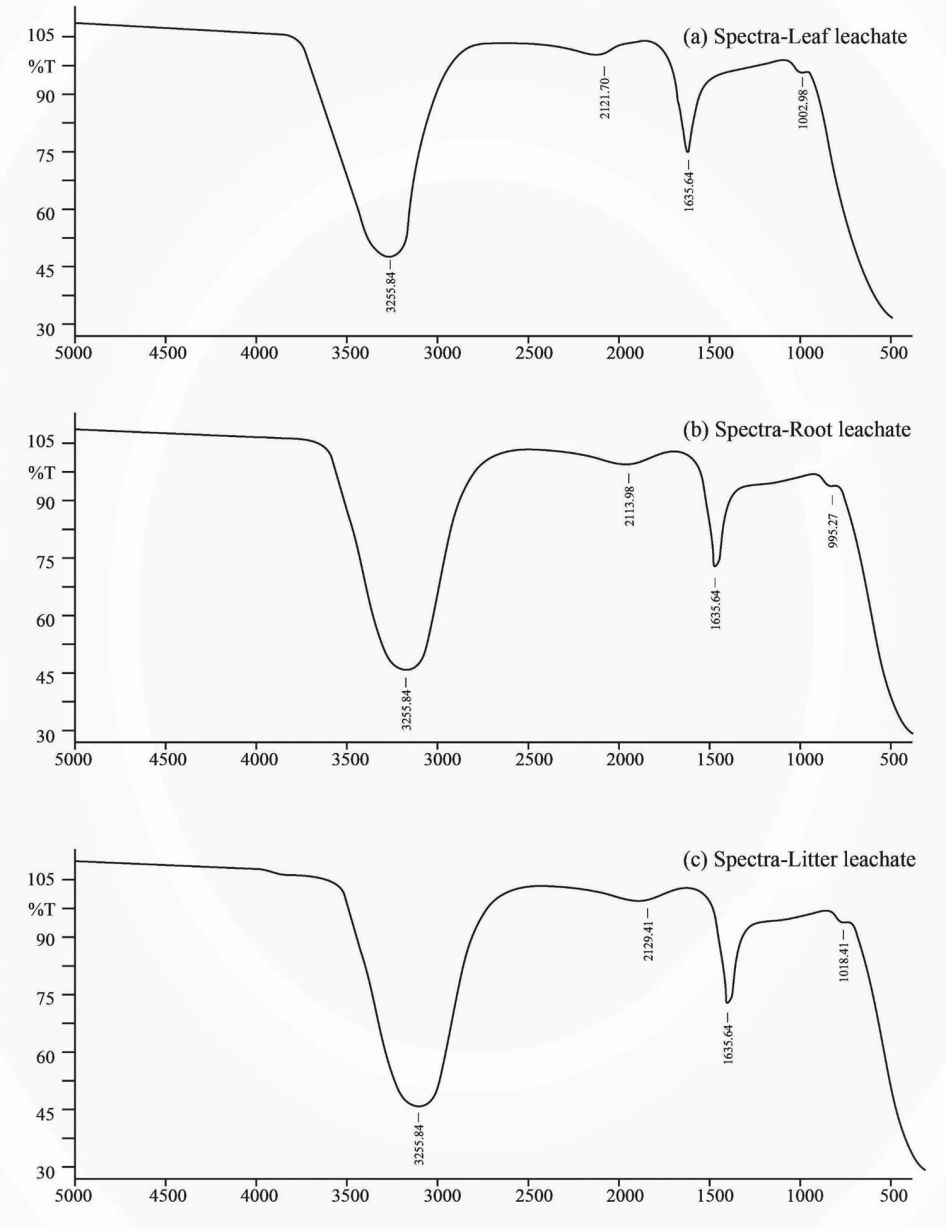
Spectra of IR analysis in *A. adenophora* fresh leaf, root and litter leachates. The names of the functional groups are given in Table 1.

Infrared spectroscopy (IR) analysis showed that all the leachates (leaf, litter, and root) of *A. adenophora* have similar functional groups, that were O-H (Hydroxyl), N–H (Amine), C≡C (Alkynes), C–H stretching (Aromatic) or C–O–C stretching (Ethers) groups (Fig. 4, Table 1). The wavenumber of the hydroxyl group was 3255.84 cm^−1^, alkynes had wavenumbers ranged from 2113.98 cm^−1^ to 2129.41 cm^−1^, amines had 1635.64 cm^−1^, and the wavenumber of C-H stretching (Aromatic) or C–O–C stretching (Ethers) varied from 995.27 cm^−1^ to 1018.41 cm^−1^ (Fig. 4, Table 1).

**Table 1:** Functional groups of chemicals found in *A. adenophora* leachates.

S.N.	FTIR peak value			Functional groups
	Leaf leachate	Root leachate	Litter leachate	
1	3255.84	3255.84	3255.84	Hydroxyl (O–H)
2	2121.7	2129.41	2113.98	Alkynes (C≡C)
3	1635.64	1635.64	1635.64	Amines (N–H)
4	1002.98	1018.41	995.27	C–H stretching (Aromatic) or C–O–C stretching (Ethers)

### Soil analysis

Soil analysis showed differences in pH, soil organic matter (OM), total nitrogen (N), available phosphorus (P) and potassium (K) between *A. adenophora* invaded and uninvaded sites. *A. adenophora* invaded soil had low pH (5.13 ± 0.06) than the uninvaded soil (5.61 ± 0.10). The concentrations of organic matter (OM), total nitrogen, available phosphorus and potassium were significantly high in the invaded soil comparing to the uninvaded soil (Table 2).

**Table 2:** Data on soil analysis (*A. adenophora* invaded and uninvaded soils).

Soil parameters	Invaded soil	Uninvaded soil	df	t-value	*p* value
pH	05.13 ± 0.06 b	05.61 ± 0.10 a	18	− 4.04	0.001
OM (%)	05.08 ± 0.15 a	04.25 ± 0.15 b		3.809	0.001
Total N (%)	00.29 ± 0.01 a	00.23 ± 0.01 b		7.163	< 0.001
K (kg/ha)	83.92 ± 3.79 a	73.07 ± 3.63 b		2.064	0.050

Soil parameters	Invaded soil	Uninvaded soil	df	Mann–Whitney U	*p* value
P (kg/ha)	00.44 ± 0.06 a	00.33 ± 0.10 b	18	20	0.023

The letters ‘a’ and ‘b’ after the mean value ± SE indicate significant differences (*p* < 0.05).

## Discussion

*Ageratina adenophora* litter showed positive effects on *E. blanda* seedling growth. Both the shoots and roots of native *E. blanda* were longer in *A. adenophora* litter treated plants compared to the control plants (Fig. 1). A similar effect was found in shoot and root biomass as well (Fig. 2). It confirms that *A. adenophora* may also have a supportive role in the growth and development of native *E. blanda.* On the other hand, shoot length and biomass were reduced by *A. adenophora* root leachate (Figs. 1, 2) but leaf leachate reduced only the biomass. From this result, it can be stated that *E. blanda* taking benefit from *A. adenophora* litter can be harmed in another way by roots or fresh leaves of *A. adenophora*. Overall, the impacts of *A. adenophora* to *E. blanda* could be negative because one component (litter) facilitates the growth whereas two-components (root and fresh leaves) have an inhibitory role. Simultaneously, the toxic effect of root and leaf leachates on chlorophyll contents (Fig. 3) may impair the light energy uptake during photosynthesis in *E. blanda*.

In the case of native *O. stellata,* the litter of *A. adenophora* increased shoot length but decreased the root length. The root leachate increased root length and biomass but decreased shoot length and biomass (Figs. 1, 2). This effect may create an abnormality in the root-shoot ratio as there is a stimulatory effect on one part (aerial or underground) and an inhibitory effect on another part (aerial or underground). The root-shoot ratio indicates overall health of plants^28,29^. Any change in normal root-shoot ratio plants would be an indication of a change in the overall health of plants^30^. The effect of the root and leaf leachates on chlorophylls of *O. stellata* was similar to the *E. blanda*. These results have confirmed that the invasive *A. adenophora* is capable to alter the root-shoot ratio and content of the photosynthetic pigments of native *O. stellata.*

There are not many validations showing the effects of *A. adenophora* on native herbs, shrubs, and tree species. Most commonly the researchers consider the crop plants and weeds as the test species. The results of some of the previous studies are in support of the findings of our study. For example, Das et al. (2018) tested the effect of this weed on seed germination and seedling growth of some crop plants and weeds^31^. The crop plants were *Triticum aestivum* and *Brassica campestris* and the weeds were *Ageratum conyzoides, Bidens pilosa, Galinsoga parviflora* and *Cyperus rotundus*. They have found that the extracts of *A. adenophora* inhibited the seed germination and seedling length of the tested plants. Similarly, Thapa et al. (2020) found that the growth and development of seedlings of native trees (*Schima wallichii* and *Alnus nepalensis*) were inhibited by *A. adenophora* fresh leaves and leaf extract^16^. Regarding the allelopathic mechanism of inhibition to native plants by invasive plants, the plant parts which are potential to produce harmful allelochemicals should be identified. Our study reveals that the fresh leaves and roots of *A. adenophora* are the parts which are the potential to produce harmful allelochemicals that can inhibit native species growth and development.

Diverse chemical compounds such as monoterpenes, sesquiterpenes, diterpenes and triterpenes, phenylpropanoids, flavonoids, coumarins, sterols, and alkaloids have been reported from *A. adenophora*. As examples, Zhou et al. (2013) have identified eleven phenolic compounds such as 7-hydroxy-8,9-dehydrothymol 9-O-trans-ferulate; 7-hydroxythymol 9-O-trans-ferulate; 7,8-dihydroxythymol 9-O-trans-ferulate; 7,8-dihydroxythymol 9-O-cis-ferulate; methyl (7R)-3-deoxy-4,5-epoxy-D-manno-2-octulosonate 8-O-trans-p-coumarate; methyl (7R)-3-deoxy-4,5-epoxy-D-manno-2-octulosonate 8-O-cis-p-coumarate; and o-coumaric acid, etc. having inhibitory effects on *Arabidopsis* seed germination^32^.

Quinic acid derivative (5-*O*-*trans*-*o*-coumaroylquinic acid methyl ester; chlorogenic acid methyl ester; macranthoin F and macranthoin G were isolated by Zhang et al. (2013) from the aerial parts of *A. adenophora*^33^*.* Similarly, thymol derivatives such as 7,9-diisobutyryloxy-8-ethoxythymol; 7-acetoxy-8-methoxy-9-isobutyryloxythymol and 7,9-di-isobutyryloxy-8-methoxythymol; 9-oxoageraphorone; (−)-isochaminic acid and (1α,6α)-10-hydroxycar-3-ene-2-one were identified by Dong et al. (2017) from roots of *A. adenophora*^34^*.* Including these chemical compounds and other monoterpenes from the aerial parts of *A. adenophora* are reported to have allelopathic potential^33,35^.

For the identification of at least the functional groups in chemicals found in the leachates of *A. adenophora,* Infrared Spectroscopy (IR) analysis was carried out in this study. The IR analysis is the most common and widely used spectroscopic technique for determining functional groups of chemical compounds existing in the leachates. The analyses had confirmed four functional groups in the leachates*.* The wavenumbers 3255.84 cm^−1^ and 1635.64 cm^−1^ indicated the presence of O–H (Hydroxyl) group and N-H (Amine) group, respectively in all types of *A. adenophora* leachates (root, fresh leaves, and litter) (Table 1). The wavenumbers ranged from 2113.98 to 2129.41 cm^−1^ found in the leachates indicated alkynes. Similarly, the wavenumbers ranged from 995.27 to 1018.41 cm^−1^ indicates the presence of C–H stretching (Aromatic) or C–O–C stretching (Ethers) in the leachates (Table 1).

Existence of these functional groups confirms that the allelochemicals identified by previous researchers are present in the root, leaf, and litter leachates and they are almost similar based on the functional groups. But, the treatments of the root leachate, fresh leaf leachate, and litter have shown different effects on the tested native species i.e., one can inhibit only aerial parts (shoot) or belowground part (root) and another can inhibit both shoot and root of native species (Figs. 1, 2). Hence, it can be expected that the concentrations of allelochemicals may vary in different parts of *A. adenophora* and the effects on growth and development of native species may depend on the concentrations. Also, further analysis of chemical compounds and their allelopathic effects should be explored to understand the exact effect on particular native species.

The study on allelopathic inhibition of the invasive species on the selected native species markedly shows that aerial and belowground parts (leaves and roots) are phytotoxic to the native plants. Fresh leaves, litter, and root extracts of *A. adenophora* were found to be toxic to the growth and development of native trees such as *Schima wallichii* and *Alnus nepalensis* in Nepal^16,19^. Phenologically, *A. adenophora* produces new leaves starting from pre-monsoon and its luxuriant growth is seen in the monsoon^16^. Meanwhile, the native species *E. blanda* and *O. stellata* also germinate during pre-monsoon to early monsoon. This coincidence has a high probability of facing allelopathic effect by the native seedlings because the rainwater washes the allelochemicals from aerial parts of *A. adenophora* and mix into the soil. Therefore, it is recommended that the whole body of *A. adenophora* should be removed before starting pre-monsoon in Nepal. This could prevent the release of allelochemicals from invasive species and mix them into the soil. If the underground parts are left unremoved there will be new sprouts. The removed plant materials should be managed properly, for example, the burial of the removed parts could be one option of the management^36^. The alternate options might be the utilization of removal parts for composting as the application of compost from *A. adenophora* may be beneficial for some crop plants^37^.

We have tested *A. adenophora* uninvaded and invaded soils to know whether there is any change caused by invasions in the soil properties. It is well known that *A. adenophora* can bring changes in soil physicochemical parameters^38^. Impacts of *A. adenophora* on soil organic matters, soil nitrogen, soil phosphorus, and soil potassium may vary with seasons, exposure, and degrees invasion level and therefore, there is no unified rule on how *A. adenophora* alter soil parameters^39^. Hence, it is suggested that the influence of particular invasive species on the soil should be studied at different invaded locations.

Soil analysis shows differences in pH, soil organic matter, total nitrogen, total phosphorus, and potassium (Table 2). *A. adenophora* had reduced the soil pH. It could be one of the mechanisms to inhibit seed germination, seedling growth, and development of native species^19^. Soils may become acidic as a result of leaching allelochemicals through rainwater. Thapa et al. (2017) expected that the reduced pH in the *A. adenophora* may reduce the seedling growth and development of native *Schima wallichii*^19^*.* Here also, it is likely that the growth and development of native *E. blanda* and *O. stellata* might have affected by reduced pH*.* Nirola and Jha (2011) have found that the soil pH in the sites having high importance value index (IVI) of *O. stellata* was 5.60^40^ which is similar to our results. Previous studies regarding the soil pH for *E. blanda* are deficient*.*

The organic matter, total nitrogen, and available phosphorus in the *A. adenophora* invaded soil were high as compared to the uninvaded soil*.* The available potassium was also increased by the invasion of *A. adenophora* (Table 2). This result was contrasting with the finding of previous studies, for instance, Thapa et al. (2017) found that there were no significant changes in these parameters in *Schima-Alnus* forest in Nepal^19^. Soil samples in this study were taken from a mixed plant community of the uninvaded sites. Therefore, during comparison of these parameters, type of uninvaded sites should also be characterized. Moreover, the contradictory results on soil parameters may mislead the readers or researchers to understand the actual pattern of invasive species interactions. Hence, it is suggested that the plant community and history of alien plant invasion as well as the degree of invasion level should be considered to know the actual pattern of changes in soil properties by invasive plants. Also, updating the soil status of native plant communities on the periodic basis would have a great significance for future predictions.

From the results, it is obvious that increasing the content of organic matter, total nitrogen and phosphorus, and potassium in the invaded sites may support the native species. However, the allelopathic effects has an equal chance to harm the native species. It is necessary to evaluate the contribution of such nutrients by invasive plants and the other allelochemical dose released by them in the invaded sites. If the dose of harmful allelochemicals released is higher than the nutrient contribution to the soil, the native plants may not take benefits from the nutrients provided by invasive alien plants.

Based on the field observation, it was difficult to predict the reasons behind association between the native species (*O. stellata* and *E. blanda*) and invasive *A. adenophora.* Our results clarify that the duration of *A. adenphora* invasion is not much longer to replace these native species but it can be expected that if the process of inhibition due to allelochemicals washed by rainwater continues, these native species might be replaced in the future. The contribution of *A. adenophora* litter might be lesser than the contribution of fresh parts (leaves) because the litters (dry leaves) are seen only during the post-winter season and the amount is relatively less, while the fresh leaves sprout enormous from pre-monsoon that remain throughout the year. The current study highlights that the interaction between *A. adenophora* and native *O. stellata* and *E. blanda* is negative and based on the results it can be expected that the population of these native shrubs would be diminished gradually in the invaded sites. Hence, regular observation and abundance measurement of the native species are recommended to confirm our prediction.

In conclusion, the impacts of *A. adenophora* to *E. blanda* is negative because one component (litter) has an advantage whereas the two-components (root and fresh leaves) have inhibitory role. Regarding the native *O. stellata,* there is a stimulatory effect of *A. adenophora* on one part (aerial or underground) and an inhibitory effect on another part (aerial or underground). This effect may create abnormality in the root-shoot ratio. The results also show that the fresh leaves and root of *A. adenophora* are the parts which are the potential to produce harmful allelochemicals that can inhibit native species. The IR analysis confirmed four functional groups in the *A. adenophora* leachates that are O–H Stretching (Hydroxyl), N-H (Amines), C–H stretching (Aromatic), and C–O–C stretching (Ethers). This result indicates that *A. adenophora* allelochemicals belong to these functional groups. Their concentrations may differ based on the vegetative parts of *A. adenophora* which may produce positive and negative effects on the growth and development of native species. Removal of the whole body of *A. adenophora* could prevent the release of allelochemicals from aerial and underground parts and mix them into the soil.

Soil analysis shows differences in pH, soil organic matter, total nitrogen, total phosphorus, and potassium between *A. adenophora* invaded and uninvaded soils. Reduction in the soil pH by *A. adenophora* can be a mechanism to inhibit the native species. Comparing the concentrations of organic matter, total nitrogen, and total phosphorus in the invaded soil with previous findings, it is suggested that the plant community and history of alien invasion as well as the degree of invasion level should be considered to know the actual pattern of changes in soil properties by an alien invasion. Monitoring the soil status frequently in the invaded regions have great significance for the future predictions. Additionally, it is necessary to evaluate the positive contribution of nutrients and the negative effects of allelochemicals released from invasive plants to estimate the net effect on the growth and development of native species.

## Materials and methods

### Test species

Two native shrubs *Osbeckia stellata* Buchanan-Hamilton ex Kew Gawler and *Elsholtzia blanda* (Benth.) Benth. were the native test species for the phytotoxicity of invasive *A. adenophora*. These species are found associated with *A. adenophora* in the invaded sites. *E. blanda* represents a member of the family Lamiaceae and *O. stellata* of the family Melastomataceae. Both reach a height up to 2 m. *E. blanda* is an aromatic plant and yields essential oil. Both species are the highly valuable medicinal plants. *E. blanda* is used in cuts and wounds, cough, choleric diarrhoea, fever, hepatitis, nephritis, pharyngitis, and cardiovascular disorders^41,42^. *O. stellata* is used in the treatment of diarrhoea, dysentery, and juice of leaves is used for scabies treatment^43^. In addition, both the species are important elements of native species composition in subtropical regions of Nepalese forests.

### Pot experiment

The native plants were grown in pots containing soil collected from the uninvaded area and treated with *A. adenophora* leachates and litter. Seeds of native *E. blanda* and *O. stellata,* and the soils were collected in February 2018 from the Takhtar Community Forest (27° 24′ 59.99′′ N and 85° 01′ 60.00′′ E, elevation: 1750–1900 masl.). The community forest is located in Thaha Municipality -9, Chitlang village of Makawanpur district, Bagmati Province, Nepal. The average annual temperature in the Chitlang village area is 16.2 °C and the average annual rainfall is 2812 mm^44^. The seeds were brought to the Central Department of Botany, Tribhuvan University, Kirtipur, and stored at 4 °C in the airtight plastic bag until use.

Polyethylene pots (size 6 × 10 cm^2^) were filled with 200 g of soil and the soil was moistened by 200 ml distilled water. The seeds of native species were placed on moist filter paper at room temperature (25 ± 5 °C) and allowed to germinate in dark. After the 7^th^ day of seed soaking, the seedlings grown were about 1 to 1.5 cm long. The seedlings of homogenous size were gently picked up and transplanted to the pots prepared. Six seedlings were transplanted to each pot containing the moist soil.

The native seedlings were grown in the pots with the following treatments (i) Control (distilled water), (ii) *A. adenophora* leachate (leachate obtained from 10 g leaves/100 ml distilled water), (iii) *A. denophora* root leachate (leachate obtained from 10 g root/100 ml distilled water), and (iv) *A. adenophora* litter (1 cm thick layer on pot surface). Each treatment had 6 replicated pots. Altogether there were 48 pots (4 treatments × 6 replicates × 2 native species = 48).

The control pots were watered (10 ml) using distilled water on an alternate day. Similarly, *A. adenophora* fresh leaf and root leachates were poured into the respective pots. The litter treatment pots were watered using 10 ml distilled water over the litter. The pots were placed in the glasshouse of the Central Department of Botany, Tribhuvn University, Kathmandu, Nepal and allowed the seedlings to grow. The positions of the pots were randomly changed regularly in the glasshouse to minimize the positional effect. The temperature of the house ranged between 20 and 38 °C and moisture between 50 and 88%. Plants were harvested on the 48^th^ days after seedling transplantation. After harvesting, the length and dry weight of roots and shoots were taken separately. The roots and shoots were dried in a hot air oven at 80 °C for 24 h for the dry weight.

### Infrared spectroscopy (IR) analysis

Infrared spectroscopy (IR) analysis of *A. adenophora* leaf, root, and litter leachates was done for determining the functional groups of chemicals present in the extract. A small quantity of each leachate was separately poured on the Attenuated Total Reflection (ATR) Diamond puck at ATR crystal of Fourier Transform Infrared Spectrometer (FTIR). The ATR is the method that allows the direct measurement of samples for FTIR. The IR spectrum was obtained using SHIMADZU IRPrestige-21, FTIR Spectrometer, Department of Plant Resource, Ministry of Forests and Environment, Government of Nepal, Thapathali, Kathmandu, Nepal. The samples were scanned 25 times with a resolution of 16 from 5000 cm^−1 ^to 500 cm^−1^ wavenumber range. The spectrum of peaks with wavenumbers was recorded.

### Soil analysis and chlorophyll estimation

*Ageratina adenophora* invaded and uninvaded soils were collected from Takhtar Community Forest, Chitlang, Makwanpur (surface soil from 5 to 15 cm). Two-line transects were made, one along each invaded and uninvaded site in the forest. Ten plots of size 1 m^2^ were sampled at each transect. Altogether 20 soil samples were collected (10 from invaded and 10 from uninvaded plots). The uninvaded plots were free of *A. adenophora* whereas the cover of *A. adenophora*,^45^ and the total nitrogen was estimated by the Kjeldahl method^46^. Estimation of phosphorus and potassium was followed by Olsen’s bicarbonate method^47^ and the Flame photometry^48^, respectively. Chlorophyll contents were estimated in the leaves sampled from the plants grown in pots under different treatments as mentioned in the pot experiment. Acetone (80%) was used for extraction and the contents were measured using the method described by Bajracharya (1999)^49^.

### Statistical analyses

The growth parameters (root shoot length and dry weight) and chlorophyll content among different treatments (root leachate, leaf leachate and litter of *A. adenophora*) were compared using one-way ANOVA. Soil parameters (pH, OM, total N and K) between the *A. adenophora* invaded and uninvaded sites were compared using independent sample t-test but the phosphorus was compared using the Mann–Whitney U test as the data was not normal. The p-value < 0.05 was considered statistically significant differences in the plant growth and soil parameters.

### Ethics and research guideline statement

Research permission including the collection of plant materials was taken from the Department of Plant Resources (DPR), Ministry of Forests and Environment, Government of Nepal, Thapathali, Kathmandu, Nepal. The experiments were conducted following relevant guidelines and regulations.
